# Bioremediation of Petroleum Hydrocarbons Using *Acinetobacter* sp. SCYY-5 Isolated from Contaminated Oil Sludge: Strategy and Effectiveness Study

**DOI:** 10.3390/ijerph18020819

**Published:** 2021-01-19

**Authors:** Yiyun Cai, Runkai Wang, Pinhua Rao, Baichun Wu, Lili Yan, Lijiang Hu, Sangsook Park, Moonhee Ryu, Xiaoya Zhou

**Affiliations:** 1 School of Chemistry and Chemical Engineering, Shanghai University of Engineering Science, Shanghai 201620, China; m040118137@sues.edu.cn (Y.C.); raopinhua@sues.edu.cn (P.R.); liliyan@sues.edu.cn (L.Y.); 15868218094@126.com (L.H.); M040119306@sues.edu.cn (X.Z.); 2 College of Civil Engineering, Kashgar University, Kashgar 844006, China; 3 State Key Laboratory of Petroleum Pollution Control, Beijing 102206, China; 4 Anji Guoqian Environmental Technology Co., Ltd., Huzhou 313000, China; 5 Department of Environmental Engineering, Sunchon National University, 255 Jungang-ro, Suncheon, Jeonnam 57922, Korea; ssp@scnu.ac.kr; 6 Division of Biotechnology, College of Environmental and Bioresource Sciences, Chonbuk National University, Iksan 570-752, Korea; Ryumh@jbnu.ac.kr

**Keywords:** biodegradation, bioremediation, TPH, *Acinetobacter* sp., 16S rDNA, response surface methodology (RSM)

## Abstract

Biodegradation has been considered as an ideal technique for total petroleum hydrocarbon (TPH) contamination, but its efficiency is limited by its application in the field. Herein, an original TPH-degrading strain, SCYY-5, was isolated from contaminated oil sludge and identified as *Acinetobacter* sp. by 16S rDNA sequence analysis. The biological function of the isolate was investigated by heavy metal tolerance, carbon, and nitrogen source and degradation tests. To enhance its biodegradation efficiency, the response surface methodology (RSM) based on a function model was adopted to investigate and optimize the strategy of microbial and environmental variables for TPH removal. Furthermore, the performance of the system increased to 79.94% with the further addition of extra nutrients, suggesting that the RSM and added nutrients increased the activity of bacteria to meet the needs of the co-metabolism matrix during growth or degradation. These results verified that it is feasible to adopt the optimal strategy of combining bioremediation with RSM to improve the biodegradation efficiency, for contaminated oil sludge.

## 1. Introduction

The oil industry inevitably produces a large amount of dense solid waste called oil sludge, which is produced during various production, transportation, and refining processes [1]. Oil sludge is exposed to biological and abiotic processes over time, such as limited microbial degradation, auto-oxidation, and volatilization, resulting in different degrees of pollution [2,3]. The pollutants in oil sludge are usually divided into inorganic pollutants such as copper, chromium and cadmium, and organic pollutants such as saturated hydrocarbons, polycyclic aromatic hydrocarbons, asphaltenes, and so on [4]. Total petroleum hydrocarbon (TPH), ranging from C15 to C36 or more, take a large fraction of the content of oil sludge [5]. Since the toxicity of oil sludge at high concentrations poses a potential risk to the environment and human health [6], it has already been listed in the US Environmental Protection Agency.

Various traditional treatment techniques for oil sludge have been proposed, like solvent extraction, chemical oxidation, landfill, natural attenuation, etc. Compared with these costly and time-consuming technologies, biodegradation is a reliable and relatively cost-effective technique to solve oil pollution [7,8]. Several studies have reported the ability of multiple organisms isolated from polluted environments, including molds, bacteria, fungi, and algae, to use petroleum hydrocarbons as their sole source of carbon and energy [9,10]. More than 70 genera and 200 species of microorganisms have been found to degrade petroleum hydrocarbons. The majority of petroleum hydrocarbon degraders are bacteria, such as *Pseudomonas* [11], *Acinetobacter* [12], *Flavobacterium* [13], and *Corynebacterium* [14], which can oxidize petroleum components through metabolic activities and be used for biological treatment of oil-contaminated areas. Many previous studies have focused on the infection and drug resistance of *Acinetobacter*, including studies on *Acinetobacter* degradation of petroleum hydrocarbons. With the continuous occurrence of oil spills worldwide, its degradation efficiency needs to be improved. Hence, it is of great significance to study and optimize the biodegradation efficiency of *Acinetobacter*.

However, bioremediation is affected by multiple variables, and it is unreliable and time-consuming to analyze, compare, and optimize the process with classical methods. As a statistical analysis method, response surface methodology (RSM) is used to overcome these shortcomings. RSM is usually developed to analyze the relationship between one or more response variables to obtain the optimal response [15,16]. The Box–Behnken Design (BBD) is one of the experimental designs of RSM. As an independent three-level factor quadratic design, it requires fewer runs and is more efficient than other designs [17]. The advantage of BBD is that it can identify potential interactions between the variables, which help to avoid experiments under extreme conditions. Although RSM has been successfully applied in many fields, such as emulsion liquid membrane processes [18], fermentation conditions [19], milk processing wastewater [20], etc., there are few reports on the use of RSM to study and optimize the removal of TPH by microbial and environmental variables. Thus, using RSM to degrade TPH has become a hot topic.

The purpose of this study was to isolate and identify the petroleum hydrocarbon-degrading bacteria in oil sludge and to solve the problem of limited efficiency of the biodegradation technique. Heavy metal tolerance, carbon, and nitrogen sources and degradation tests were used to evaluate the biological function of the isolates and their potential to degrade petroleum hydrocarbons. RSM was used to establish a quadratic equation model to optimize the isolated bacteria and environmental factors: temperature (A), pH (B), and NaCl concentration (C), and to select an optimum condition. The optimal condition and extra nutrients further promoted the bioremediation of TPH.

## 2. Materials and Methods

### 2.1. Chemicals and Media

Oil sludge samples, containing 38,236.62 mg kg^−1^ of n-alkanes, were kindly provided by the State Key Laboratory of Petroleum Pollution Control of China. The oil sludge is in a viscous solid-state, which forms agglomerates at low temperatures, and precipitates black oil at high temperatures. The sludge was stored in a refrigerator at 4 °C before the biodegradability test. Clear-up oil sludge is performed by a microwave resolution method (CJ/T221-2005) and its heavy metal elements are detected by the ICP-MS method. The average concentrations of some heavy metals in oil sludge are shown in Table 1. The ingredients of minimal salt medium (MSM, analytical grade) were as follows: 0.67 g L^−1^ NH_4_Cl, 0.8 g L^−1^ K_2_HPO_4_, 0.4 g L^−1^ KH_2_PO_4_, 0.2 g L^−1^ NaCl, 0.05 g L^−1^ CaCl_2_, 0.05 g L^−1^ MgSO_4_, 0.05 g L^−1^ FeSO_4_•7H_2_O, 0.01 g L^−1^ MnSO_4_•H_2_O, and 0.01 g L^−1^ Na_2_MoO_4_•2H_2_O [21]. The lysogeny broth (LB) medium was composed of 3 g L^−1^ beef extract, 10 g L^−1^ peptone and 5 g L^−1^ NaCl. The nitrogen-free medium was composed of 3.84 g L^−1^ citrate, 0.8 g L^−1^ K_2_HPO_4_, 0.4 g L^−1^ KH_2_PO_4_, 0.2 g L^−1^ NaCl, 0.05 g L^−1^ CaCl_2_, 0.05 g L^−1^ MgSO_4_, 0.05 g L^−1^ FeSO_4_•7H_2_O, 0.01 g L^−1^ MnSO_4_•H_2_O, and 0.01 g L^−1^ Na_2_MoO_4_•2H_2_O. All media in this study were sterilized at 121 °C for 20 min before use. The pH of the diverse media utilized in this study was 7. The standard solution UST127-TPH Mix (17 n-alkanes, 2000 mg L^−1^) was purchased from Sigma-Aldrich. The data in this study were averaged from triplicate experiments.

### 2.2. Enrichment and Isolation of the Strains

The strains from the source material, oil sludge, were enriched in a 250 mL conical flask containing 100 mL of MSM medium with 1 g of oil for seven days. The strains from the above-enriched solution were isolated, using a gradient dilution and spread plate technique. Then, different single colonies were selected and streaked on an LB plate. After multiple isolation and purification, the SCYY-5 with growth activity reaching the peak value at the soonest was screened based on the growth curve of the isolated strains.

### 2.3. TPH Biodegradation Ability of SCYY-5

To determine the degradation ability of the isolate to TPH, the isolate was cultured at 30 °C, and 150 rpm for 18 h (OD_600_:1) in the LB medium. Then, the bacterial solution was placed in a 50 mL centrifuge tube at 2500× *g* for 10 min. The centrifuged cells were washed with MSM and centrifuged for 2 to 3 times to be reserved. About 20 mL of MSM was supplemented with 1% (w/v) oil sludge in a series of 50 mL conical flasks, which were employed for biodegradation tests. The inoculation amount of bacterial liquid was 10%. Then, they were cultured at 30 °C, and 150 rpm for 10 days. The concentration of TPH in oil sludge was determined by gas chromatography (GC 2060, Shanghai Acute Instrument Co., LTD., Shanghai, China) every two days, and the TPH biodegradation efficiency was calculated.

The standard solution UST127-TPH Mix (17 n-alkanes, 2000 mg L^−1^) was used to make the standard curve (10, 20, 50, 100, 200 mg L^−1^). GC was used to analyze the degradation of TPH by the isolate after dichloromethane extraction. About 2 μL of the organic phase was injected into the GC 2060 instrument equipped with an FID detector and HP-5 capillary column (30 m × 0.32 mm × 0.25 μm, J&W Scientific, Folsom, CA, USA). The analysis conditions for GC were as follows: detector temperature, 310 °C; injector temperature, 280 °C; and carrier gas rate, 1.95 mL/min. The column temperature was kept at 50 °C for 1 min and was then ramped at 30 °C/min to 310 °C for 10 min in split mode (1:7).

### 2.4. Physiological Characterisation of the Isolated Strain

The metal salts used to prepare for Cu^2+^, Cd^2+^, Pb^2+^, Cr^3+^, Zn^2+^ (4000 mg L^−1^, 100 mL) stock solutions were 1.5125 g Cu(NO_3_)_2_·3H_2_O (241.60 g mol^−1^), 1.11 g Cd(NO_3_)_2_·4H_2_O (308.41 g mol^−1^), 0.6396 g Pb(NO_3_)_2_ (331.20 g mol^−1^), 3.0769 g Cr(NO_3_)_3_·9H_2_O (400.00 g mol^−1^), and 1.8277 g Zn(NO_3_)_2_·6H_2_O (297.48 g mol^−1^) (analytical grade) [22]. Then, the stock solutions were diluted to 0, 4, 10, 20, 30, 40, 60, 100, 150, 200, 250, 300, 400, and 500 mg L^−1^ in the LB medium. The strain SCYY-5 was cultured in the LB medium with five different metal ions: Cu^2+^, Cd^2+^, Pb^2+^, Cr^3+^, and Zn^2+^ at 30 °C, and 150 rpm to evaluate its tolerance to heavy metals. The colony numbers of bacteria were tested under the same concentration of different heavy metals by a plate counting method at log phase (18 h).

To investigate the effects of different carbon and nitrogen sources on the growth of strain, the carbon source studies were conducted using MSM as the essential medium with an equimolar concentration (20 mM) of fructose (3.60 g L^−1^), glucose (3.60 g L^−1^), sucrose (6.85 g L^−1^), lactose (6.85 g L^−1^), soluble starch (6.85 g L^−1^) and citrate (3.84 g L^−1^). The other ingredients remained unchanged [23]. They were cultured at 30 °C, and 150 rpm for nearly 40 h, and bacterial growth was monitored by turbidity measurements by measuring the absorbance of the bacterial solution at 600 nm [24,25]. The nitrogen source studies were carried out in a nitrogen-free medium, with the same equimolar concentration (20 mM) of the extra nitrogen source (2.80 g L^−1^ yeast extract, 2.94 g L^−1^ L-glutamic acid, 2.02 g L^−1^ potassium nitrate, 2.64 g L^−1^ ammonium sulfate, 1.07 g L^−1^ ammonium chloride).

### 2.5. Predictive Optimisation of TPH Degradation Based on RSM

The optimization procedure for TPH removal was conducted using BBD in Design-expert with physical-chemical parameters: temperature (A), pH (B), and NaCl concentration (C) as variables. Each aspect in the design has three different levels (−1, 0 and 1); Table 2 shows the list for each factor. A total of 17 experiments were performed in this design with TPH removal efficiency as the response. This includes 12 design diameters and 5 replication center point diameters, which were used to rule out experimental errors and fit the quadratic equation models. The statistical analysis (ANOVA) and plot response surfaces were performed using the Design-Expert 8.0 (Stat-Ease, Inc., Minneapolis, MN, USA) statistical software. Multiple regression and function models were used to evaluate the experimental data, and an F test was used to analyze the significance of the regression [17]. The following second-order polynomial equation was used to fit the experimental results and determine the relevant model terms.

(1) $$Y=\beta_{0}+\sum \beta_{i}X_{i}+\sum \beta_{ii}X_{i}^{2}+\sum \beta_{ij}X_{i}X_{j}$$

where *Y* is the predicted response; *β*_0_, *β_i_*, *β_ii_*, and *β_ij_* are fixed regression coefficients of the model; and *X_i_* and *X_j_* represent independent variables.

Meanwhile, to further improve the biodegradation efficiency, carbon and nitrogen sources were added for the biodegradation tests under the optimal conditions of RSM. According to the above physiological characteristics, the carbon and nitrogen sources with the most significant influence on the growth rate of the bacteria were selected. No additional nutrients were added under optimal conditions in (1) the control group; and the other group was (2) optimum condition + C&N sources. The other experimental conditions were consistent with the above.

### 2.6. Identification of the SCYY-5 Strain

The bacterial genomic DNA was extracted using the AxyPrep DNA isolation kit. The universal bacterial 16S rDNA primers, 27F (5′-AGAGTTTGATCCTGGCTCAG-3′) and 1492R (5′-CTACGGCTACCTTGTTACGA-3′) were used to amplify bacterial 16S rDNA [26]. The PCR product of purified strains was subjected to DNA sequencing by the sequencer ABI3730-XL. The NCBI Blast program was used to compare the spliced sequence files with the data in the NCBI 16S database (https://www.ncbi.nlm.nih.gov). The species information with the greatest similarity to the sequences to be tested was obtained, which was the identification result. Phylogenetic trees for 16S rDNA were built using MEGA 6.0 software (Arizona State University, Tempe, AZ, USA).

## 3. Results

### 3.1. Isolation and Identification of the SCYY-5 Strain

**Figure 1:**
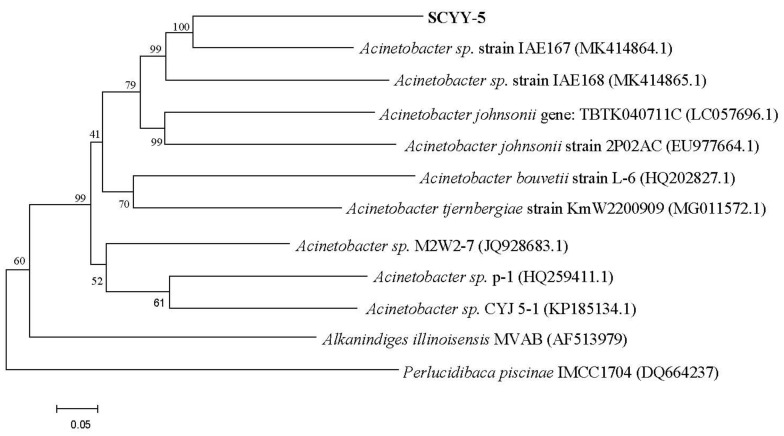
Phylogenetic trees of *Acinetobacter* sp. SCYY-5. Phylogenetic trees were constructed based on the 16S rDNA gene sequences (1241 bp) using the neighbor joining method. Phylogeny test used bootstrap method with 1000 replications.

The SCYY-5 strain with the highest growth activity was isolated from the oil sludge, which was selected from multiple isolates after multiple isolation and purification. The colonies cultured on the LB solid medium for two days were translucent, pale yellow, round, and moist with shiny edges. The isolate was identified by DNA sequencing. The sequence of the SCYY-5 strain was searched in an NCBI Blast, and phylogenetic analysis based on 16S rDNA gene sequences indicated that the SCYY-5 strain belongs to the genus *Acinetobacter* sp., as shown in Figure 1.

### 3.2. Biodegradation Ability of TPH by the Isolated Strain

The concentration of 13 n-alkanes detected in the oil sludge was 38,236.62 mg kg^−1^. The degradation ability of *Acinetobacter* sp. SCYY-5 to n-alkanes was preliminarily determined after 10 days of culture. The degradation efficiency reached 69.17% on the tenth day. The content of 13 n-alkanes decreased from 38,236.62 mg kg^−1^ to 11,788.35 mg kg^−1^ after the treatment (Table S1). It was proven that this strain can degrade TPH, but further optimization and exploration were needed.

**Figure 2:**
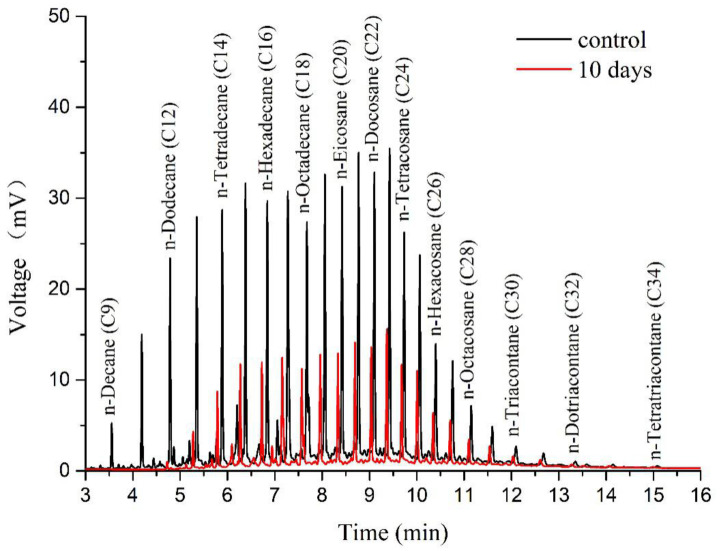
The comparison of GC profiles of TPH removal by *Acinetobacter* sp.: a control group and 10 days.

Figure 2 shows the GC profiles of TPH removal by *Acinetobacter* sp. SCYY-5. It can be seen that C14 and C28 were degraded (Table S1), and the concentration of each component decreased to some extent. Those with a moderate length (C9–C16) were lessened by 82.00%, and the long-chain alkanes (C17–C34) were spoiled by 61.76%. Interestingly, some n-alkanes such as C30 have a higher concentration than the initial concentration on the 10th day of culture.

### 3.3. Physiological Characterization of the Isolated Strain

**Figure 3:**
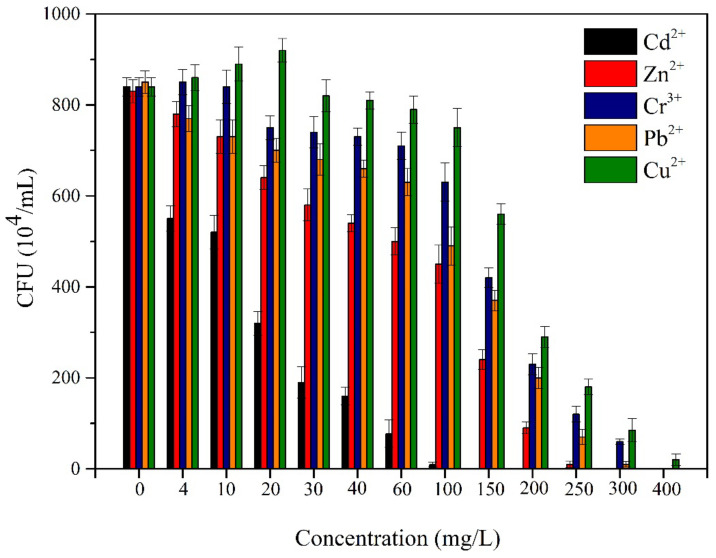
The tolerance of *Acinetobacter* sp. SCYY-5 to 5 heavy metals: Cu^2+^, Cd^2+^, Zn^2+^, Pb^2+^, and Cr^3+^. The general order of resistance of *Acinetobacter* sp. SCYY-5 to heavy metals is as follows: Cd^2+^ < Zn^2+^ < Pb^2+^ = Cr^3+^ < Cu^2+^.

The tolerance of *Acinetobacter* sp. SCYY-5 to Cr^3+^, Cu^2+^, Pb^2+^, and Zn^2+^ was slightly higher than that of Cd^2+^ (Figure 3). The growth of the strain was promoted when Cu^2+^ was less than 30 mg L^−1^, and the colony numbers further increased. Bacterial growth was also promoted when Cr^3+^ was less than 10 mg L^−1^. Pb^2+^, Zn^2+^, and Cd^2+^ had different degrees of inhibition on the isolate, and the cell survival in Cd^2+^ decreased to less than 10% at 60 mg L^−1^. The general order of resistance of *Acinetobacter* sp. SCYY-5 to heavy metals was as follows: Cd^2+^ < Zn^2+^ < Pb^2+^ = Cr^3+^ < Cu^2+^. The maximal concentration tolerated was 100 mg L^−1^ for Cd^2+^, 250 mg L^−1^ for Zn^2+^, 300 mg L^−1^ for Pb^2+^ and Cr^3+^ and 400 mg L^−1^ for Cu^2+^. Although most heavy metals can damage the cell membrane and disrupt nutrient transport, Cu^2+^ was the most tolerated metal by the isolate and Cd^2+^ was the least tolerated.

**Figure 4:**
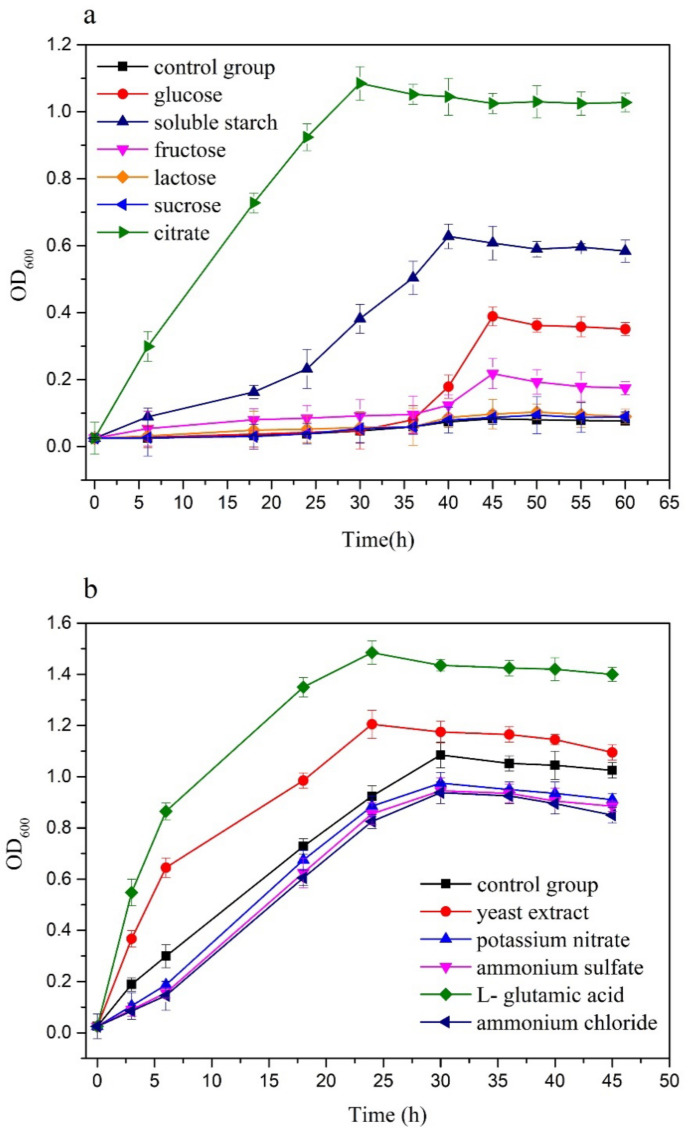
Growth profile of *Acinetobacter* sp. SCYY-5 (**a**) carbon sources, (**b**) nitrogen sources.

The carbon sources, such as glucose, sucrose, fructose, lactose, soluble starch, and citrate, were used as nutrients for bacterial growth. As shown in Figure 4a, the strain grew very slowly in the MSM medium compared with other conditions containing carbon sources. It can be considered that the presence of extra carbon sources promoted the overall growth of *Acinetobacter* sp. SCYY-5. This indicates that the carbon sources were utilized during the growth of the strain. Compared with the blank control group, it shows that the preferred carbon sources by *Acinetobacter* sp. SCYY-5 can be arranged in a sequence, as follows: sucrose < lactose < fructose < glucose < soluble starch < citrate. The maximum cell growth (OD_600_) was 1.085 under the condition of citrate as a carbon source.

Similarly, the result in Figure 4b shows the preferred nitrogen sources of the isolate in the following order: ammonium chloride < ammonium sulfate < potassium nitrate < yeast extract < L-glutamic acid. The addition of inorganic nitrogen sources did not promote the growth of the strain. The utilization rate of organic nitrogen by the isolate was significantly higher than that of inorganic nitrogen, and the maximum cell growth (OD_600_) was 1.485, with L-glutamic acid as the nitrogen source.

### 3.4. Predictive Optimisation of TPH Degradation Based on RSM

The experimental data were fitted by multiple regression in Design-Expert 8.0. The response surface test designs with the actual and predicted values obtained are shown in Table 3. The quadratic polynomial regression model equation of variables and response values obtained from the analysis is as follows:

Y = 64.03 + 0.32A + 6.16B − 0.13C + 3.17AB − 0.28AC − 0.70BC − 14.40A^2^ − 5.36B^2^ − 11.19C^2^ (2)

where Y stands for TPH removal, A is the temperature, B is the pH and C is the NaCl concentration.

To verify the reliability of the function model based on the response surface, ANOVA was done to determine the influence of various factors on the system response and their interaction. The results are shown in Table 4, *p* = 0.0248 < 0.05 indicates that the model has statistical significance. Lack of fit (*p* = 0.3081 > 0.05) is not significant, and the residual caused by random error indicates that the model fits well. Figure S1 shows the different diagnostic plots to ensure that the residuals were plotted against the predicted value. As shown in Figure S1a, the reasonable match between the standardized residuals and normal probability percentage confirms that the statistical assumptions are suitable to analyze the data. Figure S1b presents no obvious pattern, as the observed runs are randomly distributed in the range of residuals (−3, 3), confirming the adequacy of the model. The F-value is 4.84, which shows that the system response can be explained by the regression equation [27]. The fact can prove that if R^2^ is closer to 1, the stronger the prediction ability of the model [28]. Hence, the correlation coefficient R^2^ is 0.8616, and the adjusted R^2^ is 0.7596, which proves that the model fits well with the actual situation [29].

**Figure 5:**
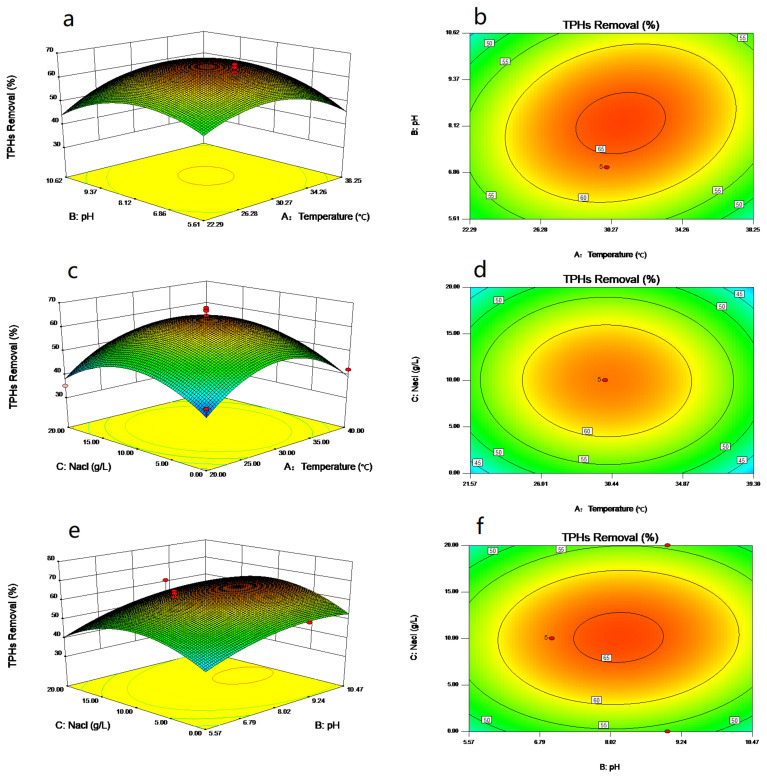
RSM response surface and contour plots for TPH removal as a function of the variables: (**a**,**b**) Temperature (A) and pH (B), (**c**,**d**) Temperature (A) and NaCl (C), (**e**,**f**) pH (B) and NaCl (C).

The Design-Expert 8.0 software was used to draw the response surface curve and contour plots for the model. The three-dimensional (3D) response surface and two-dimensional (2D) contour plots reflect the influence and interaction between any two factors of temperature (A), pH (B), and NaCl concentration (C) on TPH removal, as shown in Figure 5. For instance, as shown in Figure 5a,b, when the initial pH (temperature) value is constant, the TPH removal increases initially and then decreases with the increase in temperature (pH). The purpose of 17 groups of experiments designed by RSM is to adjust the concentration of each factor to provide an effective limiting range for BBD. The experimental results are consistent with the significance of data analysis results in Table 4. Therefore, we consider it valid that the actual value of each factor is zero.

The optimal condition (temperature = 30.77 °C, pH = 8.20, NaCl = 10.12 g L^−1^) based on RSM was predicted. The predicted value of TPH removal under the optimal condition was 65.89%, the actual experimental value was 70.29% and the residual was 4.4%. It was found that TPH removal with carbon and nitrogen sources (citrate and L-glutamic acid) under the optimal condition could reach 79.94%. The degradation efficiency of hydrocarbons increased by 9.65%. Besides, compared with the degradation efficiency of other bacteria on hydrocarbons, the SCYY-5 strain in this study had a significantly stronger degradation ability in a short time (Table 5), which can be directly applied to the treatment of pollutants.

## 4. Discussion

There are reports that have proposed that using bioremediation to solve the pollution problem of oil sludge is feasible [33,34], while the biodegradation efficiency has limited its application in the field. Many factors such as temperature, pH, salinity, etc., will have a specific influence. Therefore, this study aimed to adopt the optimal strategy of combining bioremediation with RSM to solve the problem of biodegradation efficiency.

The pollutants in oil sludge are usually divided into inorganic pollutants (copper, chromium, cadmium, salts, etc.) and organic pollutants (TPHs, PAHs, etc.), which will greatly affect the growth and activity of microorganisms. Hence, we first discussed the isolation and identification of the strain and the effect of various heavy metals on its growth. In this study, the SCYY-5 strain isolated from oil sludge provided by the State Key Laboratory of Petroleum Pollution Control was identified as *Acinetobacter* sp. Based on 16S rDNA gene sequences. According to the results of heavy metal tolerance experiments, the general order of resistance of *Acinetobacter* sp. SCYY-5 to heavy metals is as follows: Cd^2+^ < Zn^2+^ < Pb^2+^ = Cr^3+^ < Cu^2+^. The tolerance of *Acinetobacter* sp. To metal ions has been reported. Cai et al. (2019) [35] isolated an *Acinetobacter* sp. Strain from an electroplating wastewater treatment, which was resistant to Cu^2+^ and Ni^2+^, while Zakaria et al. (2007) [23] isolated the *Acinetobacter haemolyticus* strain resistant to Zn^2+^ and Cr^6+^. Compared with these reported bacteria growing in other environments, the isolates may not have the highest tolerance to a single heavy metal, but shows good resistance to Cr^3+^, Cu^2+^, Pb^2+^, Zn^2+^ but not to Cd^2+^. This can be thought of as its solubility and affinity for potential complexing agents such as organic compounds [23], and the tolerance of the isolates to Cu^2+^ was higher than the strain of Cai et al. (2019) [35], with a cell survival rate of more than 40% at 200 mg L^−1^. We used a single growth medium and did not study the tolerance value of *Acinetobacter* sp. to heavy metals in other cultures. Therefore, it can be considered that the obtained tolerance value of heavy metals is not absolute [36].

In laboratory experiments, C/N sources were added to the culture medium to evaluate the effect of various nutrients on cell yield by a turbidity measurement, during the same culture time. The result showed that *Acinetobacter* sp. SCYY-5 could quickly utilize citrate and L-glutamic acid, and exhibit high metabolic activity. It is known that *Acinetobacter* sp. cannot use carbon sources extensively, while *Acinetobacter* sp. *Y1* isolated by Liu et al. (2015) [37] used citrate and pyruvate during metabolic processes and *Acinetobacter johnonii DBP-3* isolated by Li et al. (2013) [38] was also able to utilize carbon sources (sodium citrate > glucose). This is consistent with the preference of the isolates for citrate in this study. Moreover, the isolate showed significant differences in nitrogen source utilization. Compared with the control group, there was no good cellular activity during the 50 h of growth when the inorganic nitrogen source was added. L-glutamic acid, as an organic nitrogen source, provides a mixture of peptides, which can promote bacterial growth to 1.485 (OD_600_) in 24 h of growth.

**Figure 6:**
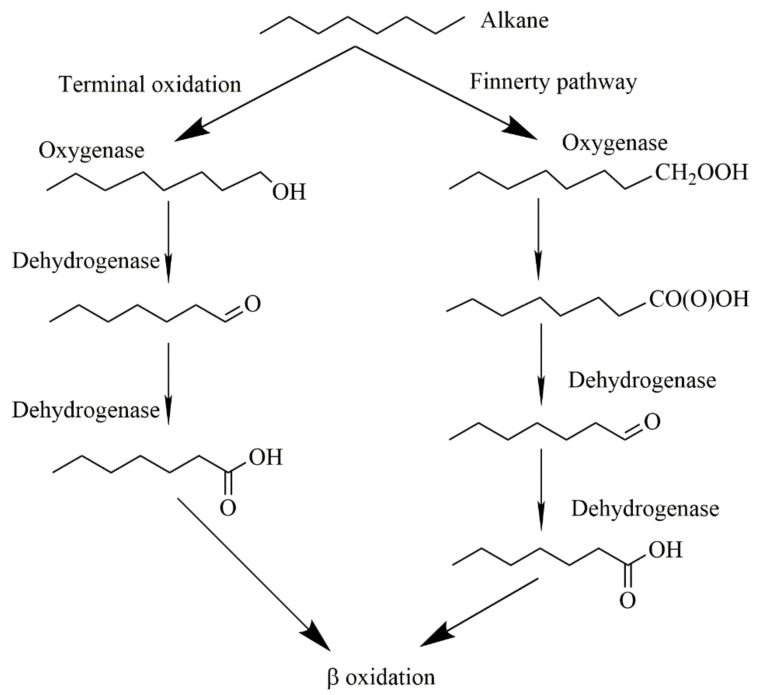
Two aerobic pathways of alkane degradation by *Acinetobacter*: Terminal oxidation and Finnerty pathway.

The preliminary degradation study indicated that the oil usually forms a thin layer in the water system, and the degree of oil dispersion partially determined the surface area of the oil that *Acinetobacter* sp. SCYY-5 could contact [39]. The bacteria were mainly active in the oil–water interface, and the increase in the available boundary area would promote biodegradation [40,41]. For bioremediation of oil sludge pollution, some studies have evaluated the ability of bacteria to degrade hydrocarbons. However, most bacteria can only use limited hydrocarbon compounds. For instance, the *Geobacillus jurassicus* isolated by Nazina et al. (2005) [42] can only grow on C6–C16, and the *Acinetobacter* sp. *BT1A* isolated by Acer et al. (2016) [43] is capable of growing on C11–C34. While the *Acinetobacter* sp. *SCYY-5* in this study showed a higher hydrocarbon degradation potential for C9–C34. The degradation efficiency of the isolate reached 69.17% on the 10th day of culture. Table 2 shows that it also has a good degradation effect on long-chain alkanes. It is generally believed that these strains can respond to petroleum hydrocarbon stress. Saturated hydrocarbons are degraded efficiently with the degradation of alkanes through terminal oxidation and Finnerty pathway [44]. Figure 6 shows two aerobic pathways of alkane degradation by *Acinetobacter*, especially for n-alkanes with more than one carbon atom, the most common degradation pathway is the terminal oxidation of alkanes. Microorganisms attack the terminal methyl groups of n-alkanes and generate primary alcohols under the action of oxygenases, which are further oxidized to aldehydes and fatty acids, and enter β oxidation [45]. Nonetheless, these biodegradation mechanisms need to be further studied and verified.

The combination of excessive biological stimuli, such as adding excessive nutrients to polluted environments or changing environmental variables, will also inhibit the growth activity of bacteria [46,47]. Therefore, replacing the traditional biodegradation technique with RSM can provide a more effective application to improve bioremediation. RSM used ANOVA to conduct statistical analysis of the model. It analyzed and optimized the relationship between bacteria and multiple environmental variables (pH, temperature, and NaCl) to obtain the best response. The results showed that the R^2^ value was 0.8616 and the adjusted R^2^ value was 0.7596. The high values of R^2^ and adjusted R^2^ reflected the success of the model prediction. The closeness of these two values indicated that the experimental results were compatible with the model [48,49]. In particular, under the optimal growth conditions (temperature = 30.77 °C, pH = 8.20 and NaCl = 10.12 g L^−1^), the degradation efficiency of TPH increased to 79.94% within 10 days with the addition of complex carbon and nitrogen sources. It is documented that bioremediation strategies with added nutrients often enhance oil degradation significantly [50]. The complex carbon and nitrogen sources could provide a more comprehensive nutritional requirement for bacteria to meet the needs of the co-metabolism matrix during growth or degradation.

## 5. Conclusions

In this study, the SCYY-5 strain that survived in the presence of high concentrations of TPH was isolated. It was identified as *Acinetobacter* sp. by 16S rDNA sequence analysis. Through the 10-day degradation experiment, it was determined that the isolated bacteria had biodegradation ability in oil sludge. TPH removal reached 69.17% on the 10th day of culture, which was an effective bacterial degradation. The optimum degradation conditions (temperature = 30.77 °C, pH = 8.20 and NaCl = 10.12 g L^−1^) of TPH were predicted by RSM. Under the optimal conditions, with the addition of citrate and L-glutamic acid, the TPH biodegradation efficiency was improved to 79.94%. The results indicated that optimal strategy is suitable and is greatly significant to bioremediate oil sludge pollution.

## Figures and Tables

**Table 1:** The average concentrations of some heavy metals in oily sludge.

Heavy Metals	Concentration (mg kg^−1^)
Cu	2116.76 ± 9.52
Cd	210.93 ± 2.37
Pb	69.85 ± 2.25
Cr	120.20 ± 5.04
Zn	1672.44 ± 0.75

**Table 2:** Summary of experimental factors and design.

Factor	Unit	−1	0	1
(A) Temperature	°C	20	30	40
(B) pH	/	5	7	9
(C) NaCl concentration	g L^−1^	0	10	20

**Table 3:** Box–Behnken design scheme with the observed and predicted response for TPH removal.

Run	(A) Temperature (°C)	(B) pH	(C) NaCl (g L^−1^)	TPH Removal (%)	Predicted Value (%)
1	30	7	10	67.07	64.03
2	40	7	0	42.25	39.17
3	30	7	10	64.87	64.03
4	30	9	20	60.91	54.20
5	20	7	0	41.23	37.98
6	30	9	0	53.46	53.08
7	30	7	10	53.51	64.03
8	30	5	20	40.11	40.49
9	20	7	20	35.20	38.27
10	20	5	10	44.44	40.98
11	40	9	10	50.46	53.93
12	30	5	0	35.44	42.15
13	30	7	10	67.97	64.03
14	20	9	10	43.32	46.95
15	40	7	20	35.11	38.35
16	30	7	10	66.75	64.03
17	40	5	10	38.88	35.26

**Table 4:** ANOVA analysis of the quadratic model.

Parameter	Sum of Squares	Degree of Freedom	Mean Square	F-Value	*p* > F	
Model	2017.66	9	224.18	4.84	0.0248	Significant
A	0.79	1	0.79	0.017	0.8995	
B	303.64	1	303.64	6.56	0.0375	
C	0.14	1	0.14	0.003	0.9578	
AB	40.32	1	40.32	0.87	0.3818	
AC	0.31	1	0.31	0.0066	0.9375	
BC	1.93	1	1.93	0.042	0.8439	
A^2^	872.66	1	872.66	18.85	0.0034	
B^2^	121.00	1	121.00	2.61	0.1500	
C^2^	527.54	1	527.54	11.39	0.0118	
Residual	324.15	7	46.31			
Lack of Fit	180.54	3	60.18	1.68	0.3081	Insignificant
Pure Error	143.61	4	35.90			
Cor Total	2341.81	16				
R^2^ = 0.8616	Adj R^2^ = 0.7596					

**Table 5:** Comparison of degradation period and degradation efficiency of hydrocarbons by different strains.

Species	Degradation Period (Day)	Degradation Efficiency (%)	References
*Pseudomonas* sp. CS-2	7	41	[30]
*Rhodococcus* sp. PG-39	7	48	[30]
*Bacillus* sp. E3	21	63	[31]
*Acinetobacter* sp. XM-02	10	74.32	[32]
*Acinetobacter* sp. SCYY-5	10	79.94	This study

## Supplementary Materials

The following are available online at https://www.mdpi.com/1660-4601/18/2/819/s1, Table S1. The content of the various hydrocarbons in oily sludge before and after the treatment; Figure S1. Diagnostic plots for TPHs removal (a) normal plot of residuals: standardized residuals versus normal probability percentage, and (b) standardized residuals plotted against predicted value.
