# Supplementary material for: Bioremediation of Petroleum Hydrocarbons Using Acinetobacter sp. SCYY-5 Isolated from Contaminated Oil Sludge: Strategy and Effectiveness Study
Source: Int J Environ Res Public Health. 2021 Jan 19;18(2):819. doi: 10.3390/ijerph18020819 (PMC7835959; doi:10.3390/ijerph18020819)
Supplement: Supplementary file 1 [file ijerph-18-00819-s001.pdf]

Supplementary:

Table S1. The content of the various hydrocarbons in oily sludge before and after the treatment.

Hydrocarbon	Concentration (mg/kg)		Removal (%)
	**Control**	**10 days**	
n-Decane (C9)	640.36 ± 2.14	194.06 ± 15.46	69.71 ± 2.31
n-Dodecane (C12)	2569.77 ± 17.45	271.84 ± 9.29	89.42 ± 0.29
n-Tetradecane (C14)	3767.35 ± 17.09	270.92 ± 15.89	92.81 ± 0.45
n-Hexadecane (C16)	7024.39 ± 40.77	1784.1 ± 16.35	74.60 ± 0.38
n-Octadecane (C18)	4798.31 ± 5.77	1229.15 ± 3.76	74.38 ± 0.05
n-Eicosane (C20)	2636.02 ± 21.94	1986.51 ± 5.42	24.64 ± 0.42
n-Docosane (C22)	6230.3 ± 49.06	2107.21 ± 10.99	66.18 ± 0.44
n-Tetracosane (C24)	5120.9 ± 11.72	1892.79 ± 6.08	63.04 ± 0.03
n-Hexacosane (C26)	2453.68 ± 17.66	542.93 ± 5.67	77.87 ± 0.39
n-Octacosane (C28)	1321.88 ± 13.81	6.87 ± 0.31	99.48 ± 0.02
n-Triacontane (C30)	337.83 ± 6.32	347.89 ± 11.24	-2.98 ± 5.25
n-Dotriacontane (C32)	648.33 ± 11.89	549.56 ± 9.28	15.24 ± 2.98
n-Tetratriacontane (C34)	687.5 ± 16.70	604.52 ± 3.74	12.07 ± 1.59
Total	38236.62 ± 58.35	11788.35 ± 31.1	69.17 ± 0.13

Figure S1

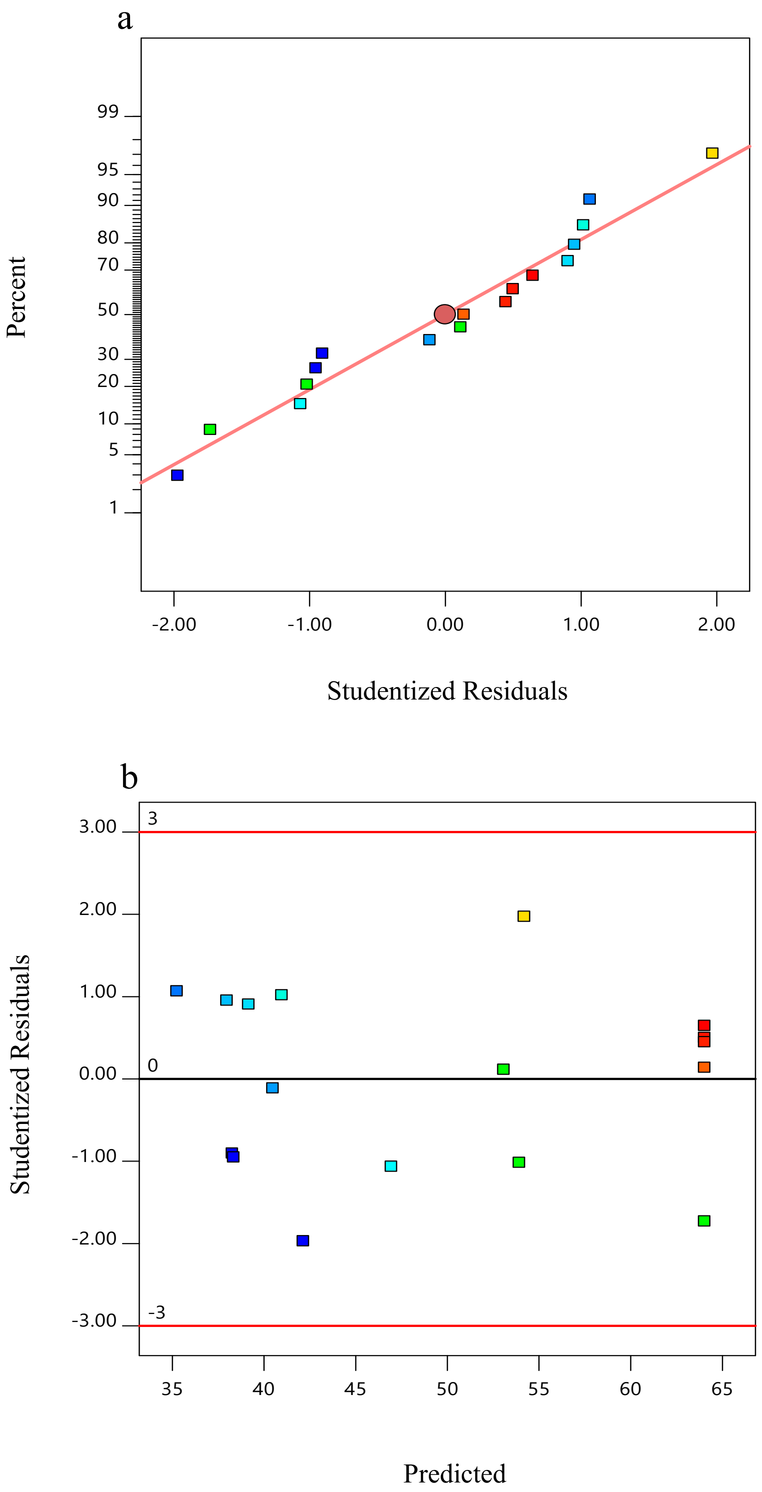

Figure S1. Diagnostic plots for TPHs removal (a) normal plot of residuals: standardized residuals versus normal probability percentage, and (b) standardized residuals plotted against predicted value.
